# Probabilistic human health risk assessment of 1,3-butadiene and styrene exposure using Monte Carlo simulation technique in the carpet production industry

**DOI:** 10.1038/s41598-022-26537-9

**Published:** 2022-12-21

**Authors:** Amir Hossein khoshakhlagh, Agnieszka Gruszecka-Kosowska, Abiodun Olagoke Adeniji, Lang Tran

**Affiliations:** 1 Department of Occupational Health Engineering, School of Health, Kashan University of Medical Sciences, Kashan, Iran; 2 Department of Environmental Protection, Faculty of Geology, Geophysics, and Environmental Protection, AGH University of Science and Technology, Al. Mickiewicza 30, 30-059 Krakow, Poland; 3 Department of Chemistry and Chemical Technology, National University of Lesotho, P. O. Roma 180, Lesotho, South Africa; 4 Institute of Occupational Medicine, Edinburgh, EH14 4AP UK

**Keywords:** Environmental sciences, Health occupations

## Abstract

Chemicals containing Volatile Organic Compounds (VOCs) are commonly used in the machine carpet production. 1,3-butadiene and styrene are main components of the carpenter’s glue used in carpet factories. Exposition to these chemicals can lead to a number of adverse health effects. This is the first study of the human health risk assessment due to inhalational exposure to 1,3-butadiene (BD) and styrene (ST) performed among workers in the carpet factories in Kashan city, Iran. The importance of the study was related with the fact of high popularity of carpet production in the South Asia countries. Inhalation exposure to BD and ST were measured based on the National Institute for Occupational Safety and Health (NIOSH) 1024 and 1501 methods, respectively. The cancerogenic risk (CR) and non-cancerogenic risk described as Hazard Quotient (HQ) values were calculated based on the United States Environmental Protection Agency (USEPA) method. The sensitivity and uncertainty analysis were performed by the Monte Carlo simulation (MCS) technique. The average concentration measured of BD and ST during work shifts of employees were 0.039 mg m^−3^ (0.017 ppm) and 12.108 mg m^−3^ (2.84 ppm), respectively. The mean ± SD value of estimated cancerogenic risk in inhalation exposure to BD and ST were equal to 5.13 × 10^–3^ ± 3.85 × 10^–4^ and 1.44 × 10^–3^ ± 2.36 × 10^–4^, respectively exceeding the acceptable risk level of 10^–6^ defined by USEPA. The average non-carcinogenic risk (HQ) values of BD and ST were equal to 8.50 × 10^0^ and 5.13 × 10^0^, respectively exceeding the acceptable risk level of 1. As the results of our studies exceeded both cancerogenic and non-carcinogenic risk values it indicates that adverse health effects due to inhalational exposure to BD and ST for workers in the machine carpet industry are very likely. To avoid negative health effects protective measures for employees in the factories should be introduced immediately and furher detailed research are recommended.

## Introduction

The development of industry, in addition to improving and increasing the level of well-being of human life^1^, has also created numerous problems for people at the global and regional levels^2^. Today, the use of chemicals in human life is inevitable. The use of chemicals in many aspects of life and economic activities has brought significant benefits and has changed the quality of human life^1^. On the other hand, these chemicals can be problematic for human health and the environment^1,2^. Air quality in workplaces has been one of the main concerns following the growth of industries in recent decades^3^. Poor air quality at workplaces has been linked to the presence of several hazardous chemicals, including the volatile organic compounds (VOCs) in the air^4^. Some VOCs, such as benzene, 1,3-butadiene, and styrene, are toxic and might evoke adverse health effects^5^. Irritation of the eyes, nose, throat, and lungs as well as damage to the liver, kidneys, and central nervous system are acute effects of VOCs’ exposure^4^. Asthma, respiratory symptoms, cardiovascular disease and different types of occupational cancers are chronic effects of VOCs’ exposure^5^. Extensive human exposure to these compounds in various industries around the world is a major issue of concern for human health^6^.

1,3-butadiene (BD) is a synthetic, colorless gas with the formula (CH_2_=CH)_2_. BD has been categorized as a human carcinogen by the International Agency for Research on Cancer (IARC) and also, the United States Environmental Protection Agency (USEPA) since 2019 consider BD as a high-priority for risk assessment^7^. Safety Data Sheet^8^ indicate that BD is an extremely flammable gas, may cause genetic effects and cancer in inhalational exposure, suspected of damaging fertility or the fetus. Exposure controls for humans regarding the standards for BD were set to be as follows^8^: ACGIH OEL TWA 2 ppm, OSHA PEL TWA 1 ppm, OSHA PEL STEL 5 ppm, NIOSH ILDH 2000 ppm; where ACGIH is American Conference of Governmental Industrial Hygienists, OSHA is Occupational Safety and Health Administration, NIOSH is National Institute for Occupational Safety and Health, OEL means occupational exposure limit, PEL means permissible exposure limit, TWA means time weighted average, STEL means short term exposure limit, ILDH means immediately dangerous to life or health. Based on the results of a cohort study, associations between inhalation exposure to BD and leukemia and bladder cancer, were found^7^. Diseases of cardiovascular system were described as the chronic effects of the exposure to BD^9^. Some metabolites of BD, such as monoepoxide, diepoxide, and epoxydiol, are suspected of causing DNA damage. Diepoxybutane (DEB), that is the most important metabolite of BD, causes adverse effects on DNA by generating reactive oxygen species (ROS) and 8-hydroxydeoxyguanosine (8-OHdG). Irritation of the eyes, nasal passage, and respiratory system as well as fatigue, with great effects on blood pressure, heart rate and damage of central nervous system have been reported as short term effects of BD^6,9^.

Styrene is a chemical with the formula of C_8_H_8_. ST is an aromatic hydrocarbon derived from benzene and has a sweet odor. Safety Data Sheet^10^ indicate that ST is a flammable liquid and vapor, causes skin irritation and serious eye irritation, is harmful if inhaled, may cause respiratory irritation and drowsiness or dizziness, is suspected of damaging the fetus, is suspected of causing cancer. Defined target organs due to exposure to BD are respiratory system, ears, and central nervous system^11^. Exposure controls for humans regarding the standards for ST were as follows^10^: ACGIH TLV TWA 10 ppm, ACGIH TLV STEL 20 ppm, OSHA PEL TWA 50 ppm, OSHA PEL STEL 100 ppm, NIOSH ILDH 700 ppm; where ACGIH is American Conference of Governmental Industrial Hygienists, OSHA is Occupational Safety and Health Administration, NIOSH is National Institute for Occupational Safety and Health, OEL means occupational exposure limit, PEL means permissible exposure limit, TWA means time weighted average, STEL means short term exposure limit, ILDH means immediately dangerous to life or health. Occupational exposure to ST affects negatively the human health, including effects on the peripheral and central nervous system (with symptoms of drowsiness, headache, and imbalance), respiratory system, and liver damage^12^. Based on the evaluation, absorption of ST is immediate via skin contact and through the lung, mainly disseminated in adipose tissue, and extensively metabolized in the body^12^. The results of Ruder et al. conducted in plastic injection industry have presented an increase in the occurrence of leukemia and lymphoma due to exposure of workers to ST^13^. Styrene has been designated as Group 2A (probably carcinogenic to humans) by the IARC in 2019^14^ and also, US National Toxicology Program (US NTP) considered ST as a carcinogenic substance^15^.

In spite of its toxic properties, both chemicals are used mainly as monomers to produce various types of polymers and copolymers such as styrene-butadiene copolymer and as a chemical intermediate in the manufacturing of some industrial chemicals^16^. The global BD market volume is over 12 million tons per year^17^. Carpet manufacturing workers are exposed to BD and ST^18,19^.

Regarding workers safety, US Occupational Safety and Health Administration (OSHA) limits exposure to BD to not more than 1 ppm (2.21 mg m^−3^) for eight hours or 5 ppm (11 mg m^−3^) for 15 min^20^. US OSHA determined the permissible exposure level (PEL) for ST to be 100 ppm and American Conference of Governmental Industrial Hygienists (ACGIH)—the Threshold Limit Value (TLV) to be 20 ppm^21^. The main exposure pathway for the BD and ST is inhalation due to volatile properties of these chemicals. One of the most reliable methods for measuring exposure is direct measurement of the concentration of the contaminant in a person's respiratory zone^9^. Risk assessment is described as determining the potential unfavorable health effects of environmental hazards^22^, as well as a tool to determine the risks of hazards in the workplace by considering the existing control measures and deciding whether to accept or not^23^.

Quantitative estimate of carcinogenic risk and non-carcinogenic risk from inhalation exposure to VOCs were developed by various agencies, such as USEPA^5^. The quantitative method proposed by the United States Environmental Protection Agency (USEPA) is an important and common method in the field of chemical risk assessment^6^. In this method, to determine the level of carcinogenic risk of exposure to chemical compounds, the cancerogenic risk (CR) index is used^6^. The use of quantitative risk assessment methods is considered by many international organizations, including the World Health Organization (WHO)^6^ and the USEPA^23^, as the basis for legislation on chemical compounds^24^.

Numerous studies have investigated the health risk of exposure to harmful chemical compounds in various industries like petrochemical^6^ and oil refinery in Iran^25^, leather, wooden furniture, printing, dyeing, garment manufacturing in China^24^, and among hospital workers in Malaysia^26^ (Table 1). Wani and Jaiswal (2012) reported that carpet weaving in Kashmir, India was related with various health hazards, not only various chemical compounds but also dust^27^Subedi and Banamala in their research revealed that carpet factory workers in Nepal in more than 50% were young women and for 44% of the carpet factory workers their families worked in the same occupation^28^. Due to the harmful work conditions in India Wani et al. 2015 pointed the necessity to introduce some provisions to protect workers’ health like masks, earplugs, first aid facilities, and proper personal protective equipment^29^. Literature search revealed that more investigations were carried on the ready carpet products and their impact on customers due to carpeted floors in their offices, schools, houses, etc.^30^.

**Table 1:** Health risk assessment research on occupational exposure related with the study.

Pollutants	Exposure	Results	References
1,3-butadiene	Petrochemical plant, Esfahan province, Iran	Definite to unacceptable risk for workers	^6^
N,N-dimethyl formamide (DMF), methyl acetate, wood dust	Leather industry, Zhejiang province, China	Severe risk for workers	^24^
Formaldehyde, xylene, styrene, toluene-2,6–diisocyanate (TDI), ethyl acetate, butyl acetate	Wooden furniture industry, Zhejiang province, China	Severe risk for workers	^24^
Formaldehyde, acetic acid, hydrogen peroxide, hydrogen sulfide, ammonia, toluene, ethyl acetate, butyl acetate	Printing and dyeing industry, Zhejiang province, China	Medium risk for workers	^24^
Fiber dust, cotton dust	Garment industry, Zhejiang province, China	Low risk for workers	^24^
Formaldehyde	Hospital workers, Selangor, Malaysia	Definite to unacceptable risk in the laboratory areas	^26^
BTEX	Carpet industry, Kashan city, Iran	Unacceptable carcinogenic and non-carcinogenic risk	^31^
Formaldehyde	Carpet industry, Kashan city, Iran	Unacceptable carcinogenic and non-carcinogenic risk	^32^
Vinyl acetate	Carpet industry, Kashan city, Iran	Unacceptable non-carcinogenic risk	^33^
Suspended particle matter	Carpet production, Kashmir, India	Unhealthy working conditions	Wani and Jaiswal (2012)
Health status	Small scale carpet factories, Bhaktapur, Nepal	Unhealthy working conditions	^28^
Health status	Carpet production, India	Unhealthy working conditions	^29^
VOCs	Oil refinery, Abadan city, Iran	Unacceptable risk values for benzene and toluene	^25^
1,3-butadiene, styrene	Carpet industry, Kashan city, Iran	Unacceptable carcinogenic and non-carcinogenic risk	This study

In addition, other research indicated that removal of these pollutants from the environment required sophisticated technologies, like biofiltration^34,35^, due to the fact the air pollutants do not occur solely and the removal method has to be sufficient for variety of chemicals at the same time^36^. Thus, based on the literature review, we stated that no study was conducted before to assess the health risk of BD and ST in carpet factories.

As all chemicals present in the air are potentially hazardous to the environment the pressure is put to control their emissions^37^ .This becomes particularly important during the occupational exposure, when chemicals concentrations and exposure time might be much higher. In some countries these permissible or recommended levels of VOCs in industry might not be in force to protect the employees and some countries might not have restrictive occupational law protecting employees at all^33^. Due to the above, in our studies we performed Human Health Risk Assessment (HHRA) based on the USEPA methodology of the inhalational exposure to evaluate the general level of risk and to recognize if the further actions will be needed regarding the health safety of investigated subpopulation of workers in the machine carpet industry. The novelty of our research was performing the HHRA based on the measured concentrations of contaminants in the indoor air on the workplaces in the carpet industry factories. As the carpet industry is very popular in South Asia countries, like Iran, this was important to perform this preliminary research, especially that best to our knowledge, such research was not performed before. Moreover, the traditional carpet production process is not performed in the factory shops with modern workers protection measures but more often in family houses in India, China, Turkey, Iran, and Pakistan, with women and children participation^38^. In our research we have used the USEPA method for calculating health risk as it can be used for various exposure scenarios, not only occupational as is the case of occupational safety agencies. In the HHRA developed by the USEPA the conservative risk assessment principle is used. It means that the slightest adverse health effects are in request. Occupational risk assessment assumes high doses and intensive exposure for workers. In the carpet production industry these conditions not always have to be as such. Workers in the carpet production in South Asia often are not typical employees as whole families might produce them in ordinary buildings and without personal protection measures. Such conditions are more like environmental exposure, which in the USEPA method after adjusting exposure scenario is also used for determining the occupational risk.Thus, based on our preliminary research in this study the same methodology can be further used for health risk assessment investigations for other susceptible subpopulations, like (pregnant) women, children, and customers in the future.

Thus, the objective of our studies was the assessment of the cancerogenic and non-carcinogenic risk for workers of machine carpet industry in Iran due to the inhalational exposure to 1,3-butadiene and styrene using USEPA methodology. The detailed objectives were as follows: (1) to determine the concentrations of BD and ST at workstations in machine carpet industry factories; (2) to investigate exposure levels, health risks, and related uncertainty due to BD and ST presence, and (3) to determine the main exposure factors affecting the risk value. The findings from the present study will provide a baseline data and scientific support needed for environmental pollution management in machine carpet production factories.

## Materials and methods

### Research area and questionnaire surveys

This study was conducted in Kashan city, Iran in 2022 to evaluate the health risk related with the inhalational exposure to BD and ST present in the air on the workstation in the machine carpet factories. Based on our previous study^33^, technological process description, and experts knowledge it was indicated that exposure to BD and ST occured in the finishing shops of carpet factories. A total of 75 employees working in finishing shops of carpet factories were included in the questionaire surveys. The research was performed in compliance with ethical principles of the Declaration of Helsinki and they were approved by the Research Ethics Committee of Kashan University of Medical Sciences, Iran (No 180IR.KAUMS.NUHEPM.REC.1401.004) and informed consent was obtained from all subjects and/or their legal guardian(s). Face-to-face interviews were performed with the employees for collecting data on demographics, past diseases, and occupational history.

### Exposure site description

Machine-woven carpets woven by carpet loom, in their raw form, are not suitable for supply to the market and delivery to the customer. The presence of dead pile threads floating behind some carpets, the weakness of the roots of the pile thread and the possibility of them coming out of the carpet, the unevenness of the carpet surface, the ugliness of the sides and tarpaulins of the woven carpet, the looseness of the warp yarns and the weft of the stalks and the possibility of them coming out of the carpet texture, the filthy and dirty surface of the carpet and the presence of defects caused by different stages of weaving are among the most important defects that can be observed in raw (unfinished) carpet. To eliminate any of the above-described defects, a finishing process consisting of several stages must be provided during the machine carpet production. As chemicals containing VOCs are used at each stage of the machine carpet finishing process, vapors of BD and ST affect workers when the glue containing styrene-butadiene copolymer is poured and discharged into the glue injection boiler, and when due to the heat the vapors from the glue pan of the sizing part, glued carpets, and from the dryer are released to employees’ workstations^19^.

### Indoor air sampling and exposure assessment

#### Sampling method

Air samples of BD and ST were collected from the worker’s breathing zone in two factories located in the Kashan city, Iran. The concentrations of BD and ST were determined in winter and during eight hours work shift (8:00 to 16:00) based on the optimized NIOSH 1024^6^ and 1501^31^ methods. Altogether 247 (3 samples for each worker and 22 blanks) indoor air samples were collected during different times in the shift (beginning, middle and the end) and these results were averaged for further calculations in the study. The samples were pumped by activated coconut charcoal (front (400 mg) and rear (200 mg) for BD and front (100 mg) and rear (50 mg) for ST) made by SKC company which was connected to the worker collar (approximately in the breathing zone) using a pump at the recommended flow rate of 0.2 L min^−1^. Personal sampling pump of model AirChek TOUCH (5–5000 mL min^−1^, SKC, Inc.) was used. The time of sampling was adjusted from 70 to 120 min based on the pretest which had been carried out to control the breakthrough volume. In the next step, in order to prevent sample loss during transfer to laboratory, both sides of the adsorbent tubes were sealed with plastic caps and placed in a cool box.

#### Sample preparation and analysis

The collected samples of BD and ST were moved to extraction vials. Desorption was carried out using 4-ml methylene chloride (99.95%) (Merck Inc., Germany) and CS_2_ for BD and ST, respectively. The samples of BD and ST were subjected to ultrasonic waves for 30 min in order to complete extraction. One microliter (1 μl) of the extracted sample was measured using GC–MS (7890 gas chromatograph, and 5975 mass spectrometer, Agilent technologies, CA, USA). Helium was applied as a carrier gas, at a flow rate of 1 mL min^−1^.

In the present study, the allowable occupational exposure limits for BD and ST vapors were calculated to be 1 ppm (2.21 mg m^−3^) and 100 ppm (425 mg m^−3^), respectively, based on the values reported by US OSHA. Given that the amount of TLV-TWA provided is assuming 8 h of work per day and 5 days of work per week. In the cases when working hours exceeded 40 h per week, the amount of TLV-TWA were modified using the Brief & Scala correction model^39^, which model is used for adjusting non usual work schedules and considers longer workdays and shorter recovery period.

### Health risk assessment

#### Cancerogenic risk assessment using the USEPA method

In the present study, the quantitative risk assessment method proposed by the USEPA was used. In this method, to estimate the carcinogenic risk of exposure to BD and ST vapors, the cancerogenic risk (CR) index was used. The value of the index for BD and ST compositions during the present study were calculated using Eq. (1)^6^:

1 $${\text{CR}} = {\text{CDI}} \times {\text{SF}}$$

where CDI is chronic daily intake (mg kg^−1^ day^−1^) and SF is slope factor (mg kg^−1^ day^−1^)^−1^. As no Inhalational Unit Risk (IUR) values were available for ST, instead of exposure concentration, we used chronic daily intake (CDI) to have the unified calculating methodology as for our chemicals slope factor (SF) values for both BD and ST were available. SF is an acceptable range in which there is a potential for a response per unit of chemical exposure over a lifetime. SF values for each carcinogenic compound are provided from toxicological databases. In our calculations we used SF values equal to 6 × 10^0^ (mg kg^−1^ day^−1^)^−1^ for BD^40^ and 5.7 × 10^–4^ (mg kg^−1^ day^−1^)^−1^ for ST^41^. Chronic daily intake (CDI) is the dose of particular pollutant taken daily averaged over exposure expressed in years. CDI values in the present study were calculated using Eq. (2)^22^:

2 $$\mathrm{CDI}=\frac{\mathrm{C }\times \mathrm{ IR }\times \mathrm{ ED }\times \mathrm{ EF }}{\mathrm{BW }\times \mathrm{ AT}}$$

where C = concentration of pollutant (mg m^−3^), IR = inhalation rate (m^3^ day^−1^), ED is exposure duration (years), EF is exposure frequency (days year^−1^), BW is body weight (kg), AT is averaging time (days). The exposure and toxicological parameters used in the study are presented in Table 2.

**Table 2:** Exposure and toxicological parameters used in carcinogenic and non-carcinogenic risk assessment in this study.

Parameter	Definition	Value	References
IR (m^3^ day^−1^)	Inhalation rate, adult male	16	USEPA 2011
EF (days year^−1^)	Exposure frequency	301.06 ± 12.87	Questionnaire
ED (years)	Exposure duration	29.66 ± 1.60	Questionnaire
BW (kg)	Body weight	74.45 ± 13.68	Questionnaire
ET (hours day^−1^)	Exposure time	10.81 ± 1.89	Questionnaire
AT (ED in years × 365 days/year × 24 h/day in hours)	Averaging time	9000	^42^
SF, mg^−1^ kg^−1^ day^−1^	Slope factor	6 × 10^–1^ for BD 5.7 × 10^–4^ for ST	^40^ ^41^
RfC (mg m^−3^)	Reference concentration from inhalation	2.0 × 10^–3^ for BD 1.0 × 10^0^ for ST	^40^

According to USEPA guidelines^31^ in our study the acceptable carcinogenic risk was set to be equal to 1 × 10^–6^ (one additional cancer risk in 1,000,000 population).

### Non-cancer health risk assessment using the US EPA method

According to the USEPA methodology for calculating the non-carcinogenic risk assessment the Hazard Quotient (HQ) is used. Hazard quotient (HQ) is the ratio between actual exposure to pollutant and its reference concentration (RfC). RfC expresses the continuous inhalation exposure concentration that will not provide adverse health effects during a lifetime. The HQ values were calculated based on the Eq. (3):

3 $${\text{HQ}} = {\text{EC}}/{\text{RfC}}$$

where EC is the exposure concentration, RfC reference concentration. The target non-carcinogenic risk value was set to be equal to 1, meaning that HQ values ≥ 1 indicate nonacceptable risk levels^43^.

Exposure concentration is the daily exposure to pollutant in the inhalational route of exposure. The exposure concentration was calculated using the Eq. 4^22^:

4 $${\text{EC}} = \left( {{\text{C}} \times {\text{ET}} \times {\text{ED}} \times {\text{EF}}} \right)/{\text{AT}}$$

where EC is the exposure concentration (mg m^−3^), ET is exposure time (hours day^−1^), ED is exposure duration (years), EF is exposure frequency (days year^−1^), AT is averaging time (ED in years × 365 days/year × 24 h/day in hours). For values of the parameters see Table 2.

### Monte-Carlo simulation and sensitivity analysis

Given that human health is followed by some uncertainties, neglecting these uncertainties may result in the loss of important information. Therefore, unrealistic and incorrect decisions can be made regarding the protection of human health. Monte Carlo simulation (MCS) is a theory based on probabilistic and statistical-mathematical approach applied to study uncertainty using random sampling of each parameter. This technique can reduce uncertainty. By determining statistical indicators or identifying its distribution function, the uncertainty degree of the output variable can be described. The general structure for determining uncertainty by the Monte Carlo technique is a combination of simulations. The calculations were conducted with 10,000 iterations, which eventually yielded the results with a degree of confidence in the range of 1 to 99%^44^. In the present study, Crystal Ball software (version 11.1.2.4, Oracle, Inc., USA) was used.

### Quality control/quality assurance (QC/QA)

In the present study, blank samples were tested in field sampling and laboratory analysis to check the levels of contamination and potential errors in the stages of sampling, transfer and measurement. The results showed that the concentration of compounds in each blank sample was less than 1% of the values measured in the original samples. The concentration values in the blank samples were also subtracted from the values found in the main samples. To determine the recovery rate of the analytes and to determine the accuracy of the measurements, the spike sample test was used. The results of the measurement accuracy showed that the percentages of analytes recovery were 87% for BD and 89% for ST.

### Consent to participate

All of the subjects had full consent to participate in the current study.

## Results and discussion

Cancerogenic and non-carcinogenic human health risk assessment was conducted in our studies due to inhalational individual exposure of carpet factories’ employees to BD and ST. Best to our knowledge, the present study was the first attempt to assess the cancerogenic and non-cancerogenic health risk in occupational exposure to BD and ST for employees in the carpet industry using USEPA method.

### BD and ST concentration

Findings from this study revealed that mean personal exposure to BD and ST was equal to 0.039 mg m^−3^ (0.017 ppm) and 12.108 mg m^−3^ (2.840 ppm), respectively. Concentrations of BD and ST determined in our studies were lower than the Occupational Exposure Limit (OEL) values recommended by the Environmental and Occupational Health Center of Iran (EOHCI). Occupational Exposure Limit Time-Weighted Average [OEL-TWA] was set to be 4.42 mg m^−3^ (2 ppm) for BD^6^ and 86 mg m^−3^ (20 ppm) for ST^43^. The temperature of the glue injection boiler and dryer and the percent of styrene-butadiene copolymer entering the boiler impacted the concentration of BD and ST. For instance, in some process the proportion of monomers of 70% to 75% of butadiene versus 20% to 25% of ST are typically used. While, in other process the proportion of monomers of 55% to 65% of butadiene versus 45% to 35% of ST are mainly used. These differences could influence the concentration of BD and ST emitted from the source.

### Cancerogenic risk (CR)

**Figure 1:**
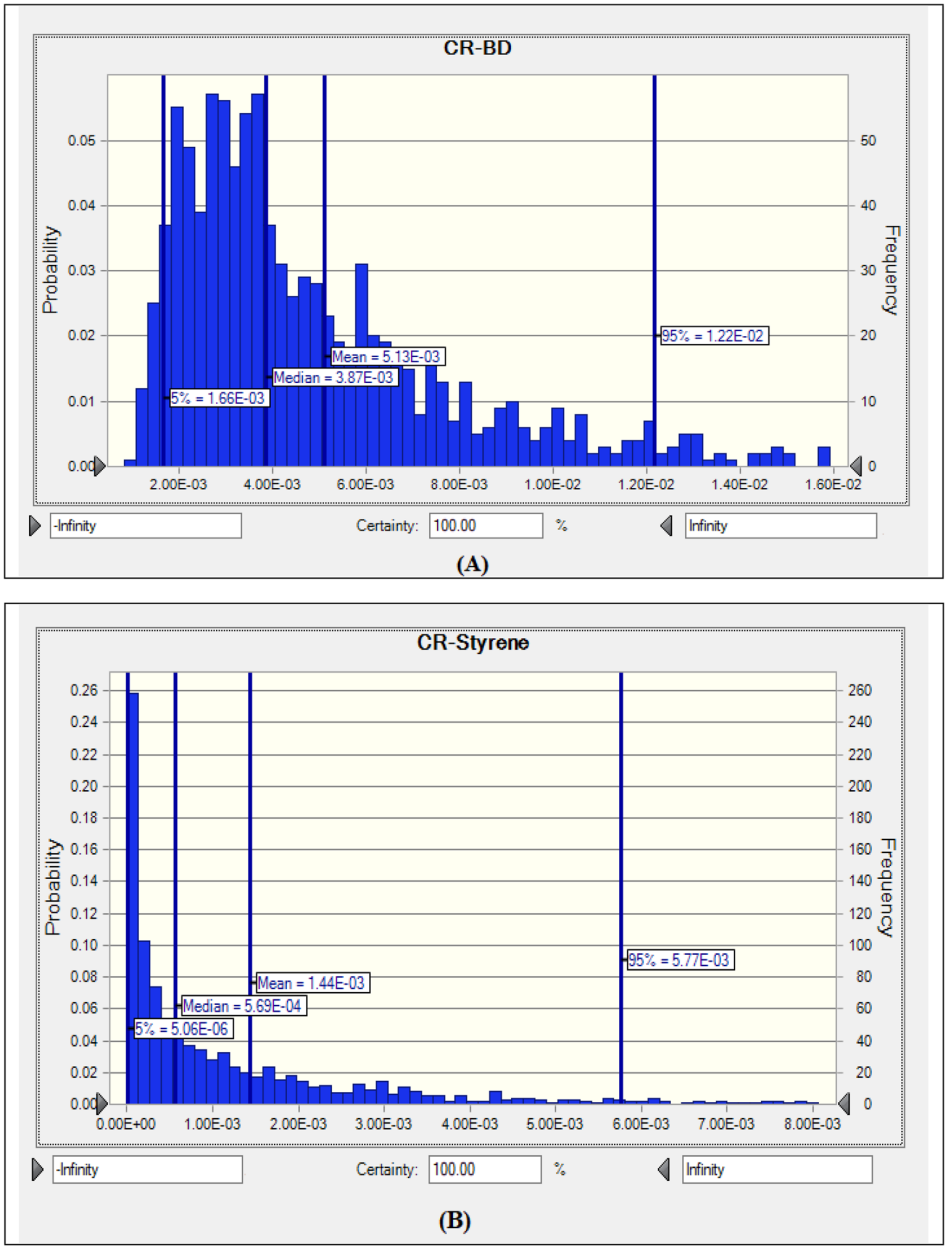
The cancerogenic risk for (**A**) 1,3-butadiene (BD), (**B**) Styrene (ST).

Cancerogenic risk assessment (CR) was applied to determine the cancer risk due to BD and ST exposure. Based on the International Agency for Research on Cancer (IARC), BD is designated as a group 1 human carcinogen (carcinogenic to humans) and ST is listed as a group 2A human carcinogen (probably carcinogenic to humans)^43^. The mean ± SD values of estimated cancer risk in inhalation exposure to BD and ST were 5.13 × 10^–3^ ± 3.85 × 10^–4^ (5.13 additional cases of cancer per 1,000 workers exposed) and 1.44 × 10^–3^ ± 2.36 × 10^–4^ (1.44 additional cases of cancer per 1,000 workers exposed), respectively (Table 3). According to the results, the 95th percentile of the carcinogenic risk values obtained for BD and ST (1.22 × 10^–2^ and 5.77 × 10^–3^, respectively) exceeded recommended by USEPA the acceptable risk level (1 × 10^−6^), meaning a significant cancerogenic risk for the exposed employees. The probability distributions of the cancerogenic risk for the pollutants (mean, median, 5th percentile, and 95th percentile) are depicted in Fig. 1. The mean and median values of BD and ST were higher than the USEPA acceptable level of 1 × 10^–6^. The cancerogenic risk values calculated for 5% of the high-risk population for BD and ST were 1.22 × 10^–2^ and 5.77 × 10^–3^, respectively. Even for BD the 5% of low-risk population, the cancerogenic risk values was 1.66 × 10^–3^ (1.66 additional cases of cancer per 1,000 individuals exposed). These findings revealed that occupational exposure through inhalation to BD and ST posed potential cancer risks for workers of finishing shops in carpet factories.

**Table 3:** Carcinogenic risk (CR) and probability distribution of BD and ST in the inhalation exposure to the employees in carpet industry.

Pollutant	CR (unitless)			
	Min	Max	Mean ± SD	P95
1,3-butadiene (BD)	8.55 × 10^–4^	3.38 × 10^–2^	5.13 × 10^–3^ ± 3.85 × 10^–4^	^a^1.22 × 10^–2^
Styrene (ST)	6.17 × 10^–8^	2.58 × 10^–2^	1.44 × 10^–3^ ± 2.36 × 10^–4^	^a^5.77 × 10^–3^

^a^The lifetime cancer risk value exceeds the acceptable level (10^−6^); P95 – 95th percentile.

Sadeghi-Yarandi et al. conducted a study in health risk assessment of inhalation exposure to 1,3-butadiene in a petrochemical plant. Their results showed that carcinogenic risk due to 1,3-butadiene at all investigated units was higher than the acceptable level^6^. The health risk of ST exposure in respiratory zone of workers in an electronics industry assessed by Mohammadyan et al.^45^ revealed the average CR value equal to 1.4 × 10^−3^, which was higher than the acceptable carcinogenic risk level. Also, carcinogenic risk values in the inhalation exposure of ST exceeding the acceptable level were reported in the other industries: motor and motorcycle, repair services, ship and boat building, basic chemical and plastic products manufacturing^15^, petrochemical industry^43^, and urban environment^46^.

### Non-cancerogenic risk (HQ)

**Figure 2:**
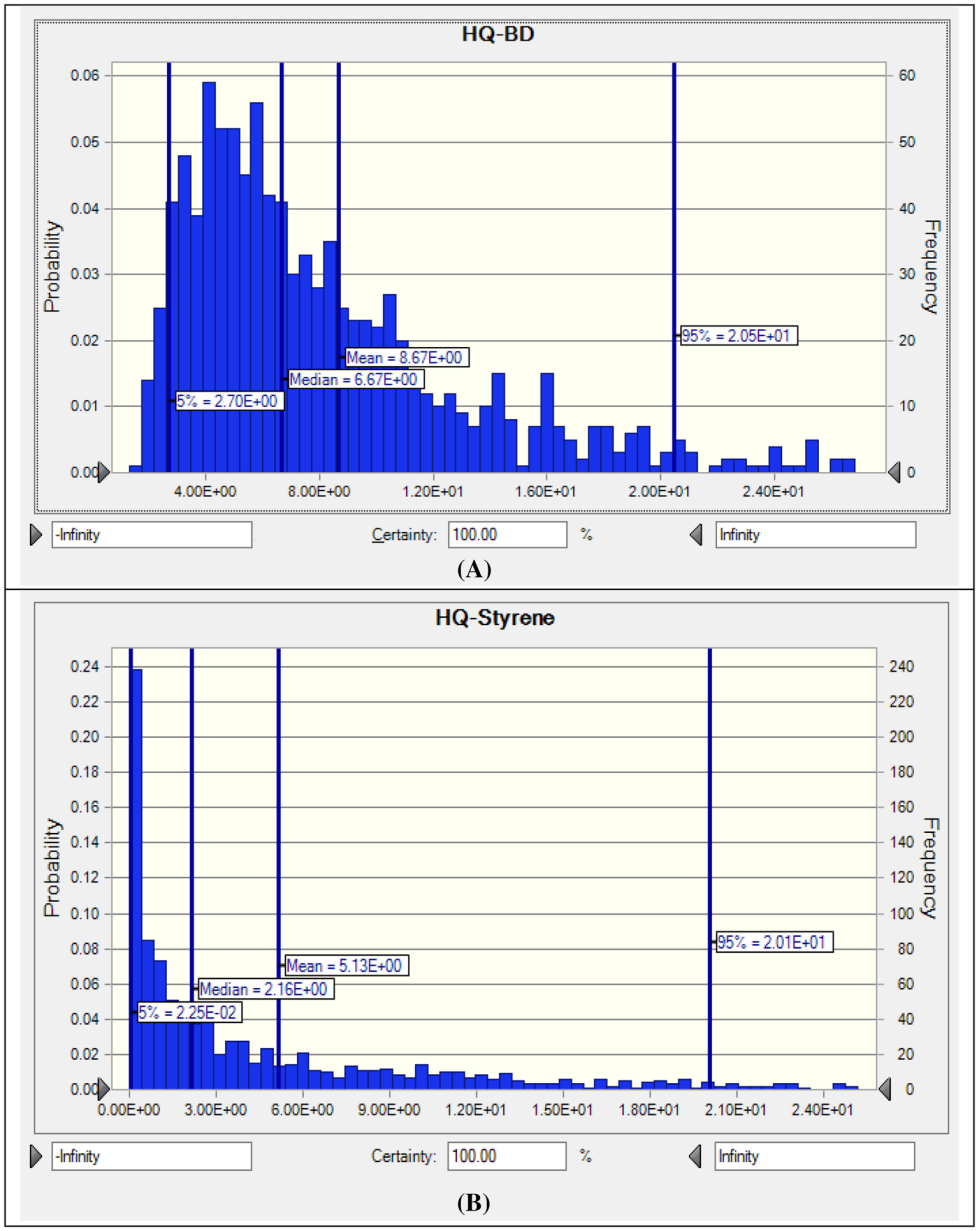
The non-cancerogenic risk for (**A**) 1,3-butadiene (BD), (**B**) Styrene (ST).

Non cancerogenic risk in the inhalation exposure to BD and ST was calculated and presented as hazard quotient (HQ) values in Table 4. Our research revealed that all calculated non-cancerogenic risk values for BD and ST were higher than the acceptable risk level of 1, except minimum ST concentration. The probability distributions of the non-cancer risk for the pollutants (mean, median, 5th percentile, and 95th percentile) are presented in Fig. 2. The mean and median risk values for BD and ST were higher than the accepatable non-carcinogeinic risk value (HQ = 1). The non-cancerogenic risk values calculated for 5% of the high-risk population for BD and ST were equal to 2.15 × 10^1^ and 2.01 × 10^1^, respectively.

**Table 4:** Non-carcinogenic risk (HQ) values and probability distribution of BD and ST in the inhalation exposure to the employees in carpet industry.

Pollutant	HQ (unitless)			
	Min	Max	Mean ± SD	P95
1,3-butadiene (BD)	1.33 × 10^0^	5.78 × 10^1^	8.67 × 10^0^ ± 6.63 × 10^–1^	^a^2.05 × 10^1^
Styrene (ST)	1.73 × 10^–4^	5.25 × 10^1^	5.13 × 10^0^ ± 7.16 × 10^–1^	^a^2.01 × 10^1^

^a^The non-carcinogenic risk value exceeds the acceptable level (HQ > 1); P95—95th percentile.

Mohammadyan et al., in risk assessment of ST exposure in an electronics industry in Iran^45^, revealed that non-cancerogenic risk values for ST were higher than the acceptable level of 1 in all industrial units^45^, which is consistent with the results of the present study. The research of Yimrungruang et al.^47^ on the health risk assessment of gas service station workers to VOCs exposure indicated that the mean ST concentration was equal to 2.4 × 10^–3^ mg m^−3^ and calculated non-cancerogenic risk value was < 1^47^, which is different from this study. As a direct relationship exists between the non-cancerogenic risk value for particular chemical and its concentration, it might be the most important factor of the calculation variations in these studies. For instance, the mean inhalation exposure to ST in the current study was 12.108 mg m^−3^, which is vastly higher than in the Yimrungruang et al. studies which was reported to be 2.4 × 10^–3^ mg m^−3^^47^. This has resulted that in the present study the mean non-cancerogenic risk values were so many times higher than in the Yimrungruang et al. study^47^.

Sadeghi-Yarandi et al., reported high HQ value for BD exposure in a petrochemical plant^6^. Furthermore, studies on BD and ST industrial exposure in microenvironments like residential homes, in-office, in-vehicle^9^, motor and motorcycle, repair services, ship and boat building, basic chemical and plastic products manufacturing^15^, and petrochemical industry^43^, reported existing risk, thus application of engineering control methods to decrease occupational exposure to BD and ST seems to be necessary.

### Sensitivity analysis

The sensitivity analysis by the Crystal Ball software was applied to identify factors influencing the most on the calculated risk values.

**Figure 3:**
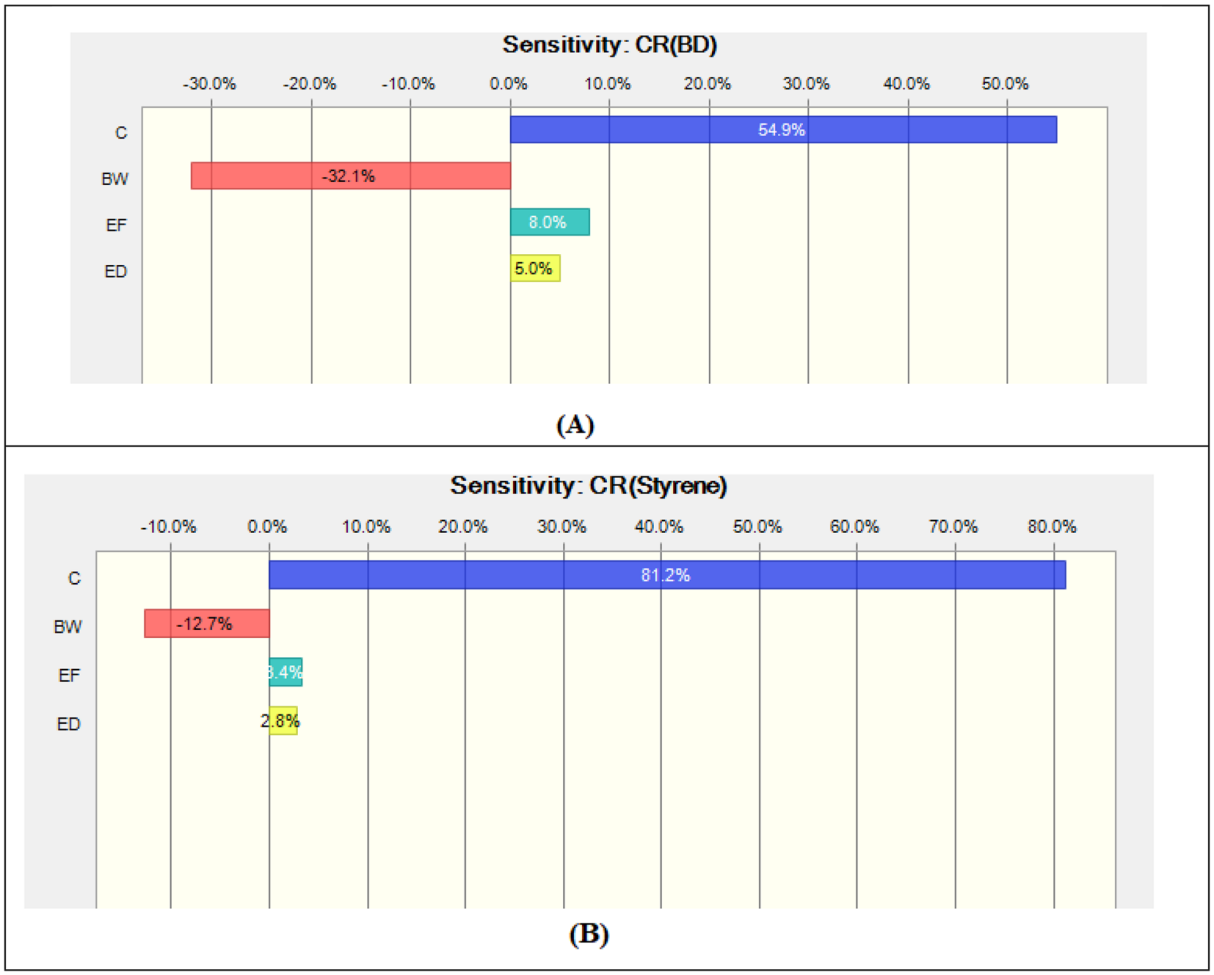
Sensitivity analysis of the cancer risk (CR) for (**A**) 1,3-butadiene, (**B**) styrene.

**Figure 4:**
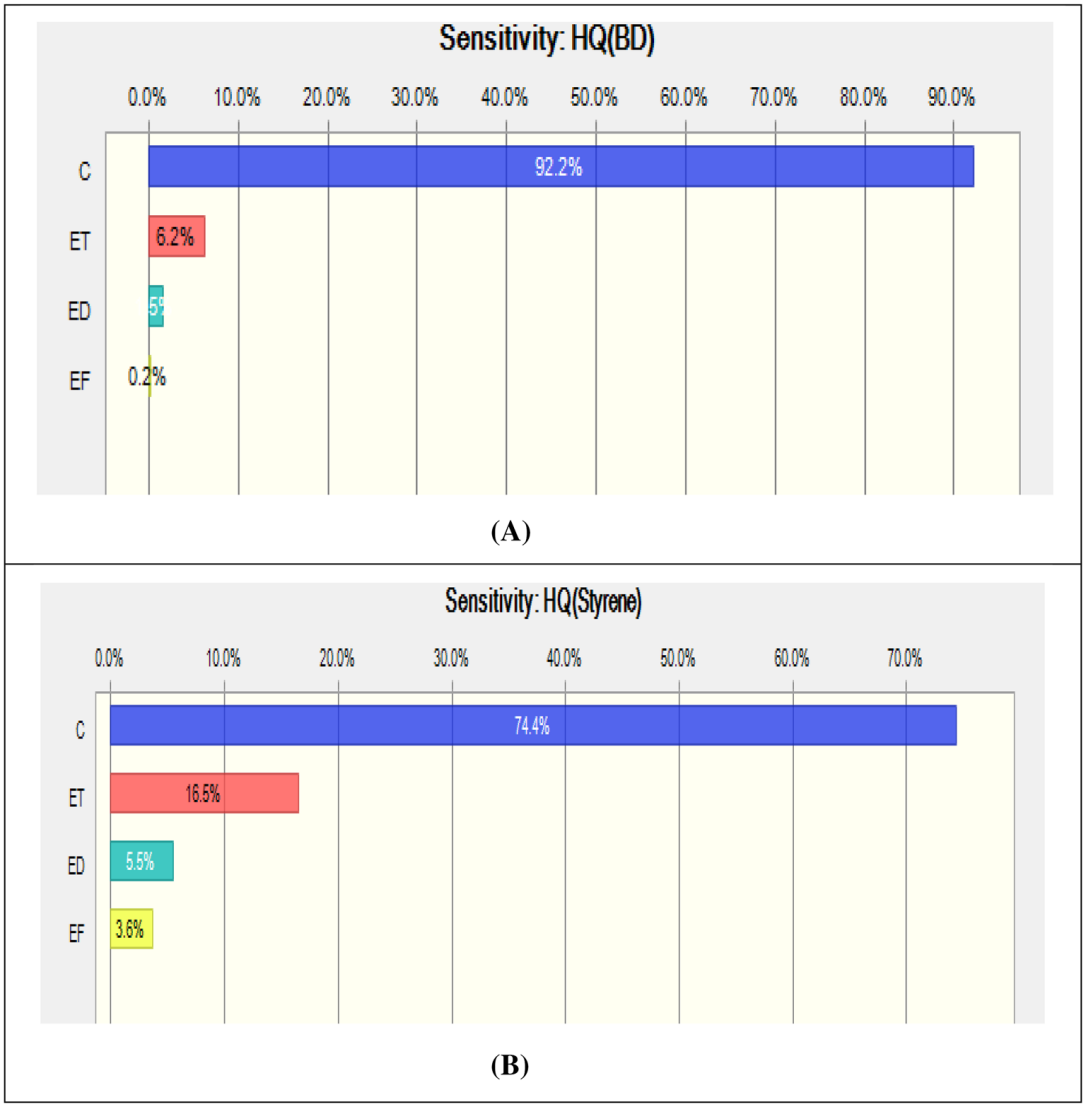
Sensitivity analysis of the non-cancer risk (HQ) for (**A**) 1,3-butadiene, (**B**) styrene.

The parameter influencing the most on the carcinogenic risk values in inhalation exposure pathway was revealed to be the pollutant concentration. Pollutant concentration affected the calculated risk values in 54.9% for BD and in 81.2% for ST (Fig. 3). The second important factor was revealed to be the exposure frequency with the values of 8% for BD and 3.4% for ST. The third important factor was the exposure duration with the values of 5% for BD and 2.8% for ST. The body weight was revealed to have the negative impact on the calculation of the carcinogenic risk values. In the case of non-carcinogenic risk values in the inhalation exposure to BD and ST, the most significant variables were: the pollutant concentration (92.2% for BD and 74.4% for ST), exposure time (6.2% for BD and 16.5% for ST), exposure duration (1.5% for BD and 5.5% for ST), and exposure frequency (0.2% for BD and 3.6% for ST) (Fig. 4).

Based on the obtained findings it should be highlighted that BD and ST concentrations need to be controlled in the workplaces as they are major influencing factors of the potential risk. Also, inhalation exposure to BD and ST should be controlled via engineering control measures as well as the exposure time of the workers should be decreased via management controls. Also, biomonitoring of the metabolites of these pollutants in the biological fluids is suggested for the future research.

### Uncertainty in the risk assessment analysis

As the uncertainty is an inherent component of the quantitative risk assessment in our studies, we considered its three components. Regarding the environmental part, uncertainty was related with performed measurements. First aspect was the number of samples collected from the worker’s breathing zone in the carpet production industry for establishing the range of inhalational BD and ST concentrations. Secondly, samples were taken only during winter season, so it is not known at this stage of the research if these concentrations were only seasonal or could be representative for the whole year. Regarding the population part, we polled 75 male employees working in finishing shops and based on these results population data for HHRA calculations were obtained, like body mass and exposure rates: duration, frequency, and time. Regarding the toxicological part, for non-carcinogenic risk calculations in our research we used the inhalational exposure pathways expressed by the exposure concentration (EC) parameter as RfC values were available in toxicological databases for both, SD and ST. In the case of carcinogenic risk, we used chronic daily intake (CDI) parameter, instead of EC, because for inhalational unit risk (IUR) needed in further calculations values were missing for ST. In carcinogenic risk analysis, as slope factor (SF) values were available for both BD and ST (IUR value was available only for BD), we used exposure pathway expressed by calculating CDI to have the consistency between BD and ST in carcinogenic risk calculations. However, we have used the lowest available SF value for ST obtained from scientific literature^41^ in accordance with the conservative risk assessment principle. SF value for ST used in our studies did not appear in the toxicological databases as the result of the lack of the agreement on confirmed SF and/or IUR value for ST. In accordance with the used conservative risk assessment principle, our risk results might be overestimated as they were prone to human health protection purposes. Expanded and more detailed future research would allow for more reliable risk calculation based on higher number of participants, different sex and age of participants, higher frequency, and longer time of air pollutants measurements in the future studies.

### Limitations and future perspectives of the study

As it was underlined earlier, best to our knowledge, this was the first studies of the health risk assessment during the process of carpet production. Thus, comparison of our results with the other research is rather limited. The results of a study by Yarandi et al.^6^, the mean cancerogenic risk (CR) of BD among the petrochemical plant workers was 2.71 × 10^–3^, which exceeded the acceptable risk value (10^–6^) recommended by the USEPA^43^. The average carcinogenic risk value of ST for workers of electronics industry in Iran was equal to 1.4 × 10^–3^ exceeding the acceptable level of USEPA^45^. The results of Ahmadi-Moshiran et al.^43^ studies in Iran, indicated that the cancerogenic risk (CR) values for BD and ST in a petrochemical industry were also higher than the acceptable level of 10^–6^ as well as the mean HQ values of non-carcinogenic risk for BD and ST were 4.04 and 0.19, respectively exceeding the acceptable risk level of 1. In the study by Hahm al.^15^, the non-carcinogenic risk (HQ) values for ST in motor vehicle, motorcycle maintenance, repair services, ship and boat building, basic chemical and plastic products manufacturing industries were higher than the acceptable risk level of 1 recommended by the USEPA.

Regarding the toxicological data in the case of BD both RfC and SF values were available in the Integrated Risk Information System (IRIS) database^40^. In the case of ST only RfC value was confirmed in the toxicological databases. In the case of the SF value for ST we used the value available in other research^41^, having in mind that so far there is no one agreed value. Banton et al. 2019^48^ reported that RfC value for ST for general population based on ototoxicity was estimated to be 6 ppm (about 25 mg m^3^). However, as this proposed RfC value was not time-adjusted from the worker occupational exposure limit of 20 ppm, it must be considered that other endpoints might occur at lower exposure duration if based on worker data^48^. Toxicological values extrapolated from non-ototoxic endpoints from animal studies may identify health values lower than those estimated on occupational ototoxicity when dose-duration adjustments, especially that application of appropriate AFs is required^48^. Also, having in mind that regarding the cancerogenic risk assessment of ST researchers are leaning to the Margin of Exposure (MOE) approach instead of the dose–response model^48^, in this study we used the existing SF value for ST to have the consistency with the applied risk calculation methodology. We have this point in mind and in the future research it is planned to apply other risk assessment models using Margin of Exposure (MOE), Point of Departure (POD), or Mode of Action (MOA), depending on the trends in the new generation risk assessment methods^49^. As the HHRA aim is to indicate the lowest exposure at which risk of the adverse health effects might occur according to the conservative risk assessment principle, in our calculations we have used the lowest SF value available from research to have the idea of the risk values, especially because this research was not performed before. Banton et al.^48^ also reported that in the fiber-reinforced polymer composites industry, HQ values for workers in open-mold processes without any respiratory protection exceeded the acceptable risk level of 1. Non-carcinogenic risk values were reduced < 1 when appropriate respiratory protection measures were used in open-molding processes, indicating that respiratory protection should be required by risk management measure for high workplace exposures^48^.

Our research revealed the need of the continuation of this study. We investigated only some of VOCs, namely BD and ST. However in our research only men were investigated, while women and even children, who often work in such industries in Asian countries, were not investigated. Regarding the chemical nature of the substances used in the carpet industry also customers seem to be affected with the vapours that can be released from the new carpet products^50^. The recent research indicate the need for intensive research in the context of the consumer health safety as carpets are presumed an exposure source of many compounds like emerging contaminats, perfluoroalkylated substances (PFAS), volatile organic compounds (VOCs), and semi-volatile organic compounds (SVOCs), especially that mechanisms like abrasion, diffusion, partitioning to airborne particles and settled dust are not well know^51^. Biomonitoring research on exposed population would be an important part of the further investigations^52^.

## Conclusion

Our research were the first studies on occupational inhalational exposure to 1,3-butadiene (BD) and styrene (ST) in the finishing shops of the carpet production industry in Kashan city, Iran. Using the USEPA Human Health Risk Assessment methodology carcinogenic (CR) and non-carcinogenic (HQ) risk values were calculated. The concentrations of BD and ST in the respiratory zone of the employees were lower than the permissible occupational exposure limits, 4.42 mg m^−3^ (2 ppm) for BD (6) and 86 mg m^−3^ (20 ppm), and were equal to 0.039 mg m^−3^ (0.017 ppm) and 12.108 mg m^−3^ (2.840 ppm), respectively. Non-cancerogenic risk (HQ) values for BD and ST were higher than the acceptable level of 1 indicating the possibility of adverse health effects to occur. The mean HQ values for the carpet industry workers were equal to 8.67 × 10^0^ for SD and 5.13 × 10^0^ for ST. Cancerogenic risks (CR) values calculated for BD and ST were higher than acceptable level of 10^–6^. The mean CR values for employees were equal to 5.13 × 10^–3^ for SD and 1.44 × 10^–3^ for ST. The sensitivity analysis revealed that BD and ST concentrations measured in the employees’ respiratory zone were the most significant parameter in the total value of both carcinogenic and non-carcinogenic risk. The application of technical and engineering control measures is recommended to reduce employees’ risk including exposure time reduction performed by management controls. More detailed research, including other susceptible populations and biomonitoring analyses of the metabolites of BD and ST in the biological fluids are required and justified for future research based on our preliminary investigations.

## Data Availability

The data sets analysed during the current study are available from the corresponding author upon reasonable request.

## Abbreviations

8-OHdG: 8-Hydroxydeoxyguanosine
ACGIH: American Conference of Governmental Industrial Hygienists
AT: Averaging time
ATS: American Thoracic Society
BD: 1,3-Butadiene
BW: Body weight
C: Concentration of pollutant
CDI: Chronic daily intake
CR: Carcinogenic risk
DEB: Diepoxybutane
DNA: Deoxyribonucleic acid
EC: Exposure concentration
ED: Exposure duration
EF: Exposure frequency
HHRA: Human Health Risk Assessment
HQ: Hazard quotient
IARC: International Agency for Research on Cancer
ILDH: Immediately Dangerous to Life or Health
IR: Inhalation rate
IRIS: Integrated Risk Information System
IUR: Inhalational unit risk
MOA: Mode of action
MOE: Margin of exposure
MCS: Monte Carlo simulation
NIOSH: National Institute for Occupational Safety and Health
OEL: Occupational exposure limit
OSHA: Occupational Safety and Health Administration
P95: 95Th percentile
PEL: Permissible exposure limit
PFAS: Perfluoroalkylated substances
POD: Point of departure
QC/QA: Quality control/quality assurance
RfC: Reference concentration
ROS: Reactive oxygen species
SD: Standard deviation
SDS: Safety data sheet
SF: Slope factor
ST: Styrene
STEL: Short term exposure limit
SVOCs: Semi-volatile organic compounds
TLV: Threshold limit value
TWA: Time weighted average
US NTP: United States National Toxicology Program
USEPA: United States Environmental Protection Agency
VOCs: Volatile organic compounds
WHO: World Health Organization
